# Stabilisation of oil-in-water emulsions with non-chemical modified gelatinised starch

**DOI:** 10.1016/j.foodhyd.2018.03.002

**Published:** 2018-08

**Authors:** Miroslaw M. Kasprzak, William Macnaughtan, Stephen Harding, Peter Wilde, Bettina Wolf

**Affiliations:** a School of Biosciences, University of Nottingham, Sutton Bonington, Loughborough, LE12 5RD, UK; b Quadram Institute Bioscience, Norwich Research Park, Colney, Norwich, NR4 7UA, UK

**Keywords:** Non-chemically modified gelatinised starch, OSA starch, Emulsion, Stability, Sedimentation coefficient

## Abstract

In this research, stabilisation of oil-in-water emulsions with non-chemically modified gelatinised starch is presented. Thus far only octenyl succinic anhydride (OSA) modified gelatinised starch has been known to adsorb at emulsion droplet interfaces, acting as emulsifiers. Screening a range of commercially available food starches revealed that a non-waxy rice starch, a waxy rice starch and the waxy maize starch PRIMA600 showed oil-in-water emulsifying ability following gelatinisation. The microstructure of emulsions formulated with 20% oil and 1% starch was stable for at least 3 months. Thermal, crystallinity and molecular property analyses as well as amylose and protein content revealed no obvious link to this property. Nevertheless, this research has provided the food industry with exciting results for the formulation of clean label emulsions. Moreover, it presents a concept for oral release food emulsions with destabilisation via salivary amylase digestion of the stabilising starch emulsifier.

## Highlights

• Non-chemically modified gelatinised starches screened for emulsifier functionality.
• High shear overhead processing of oil-in-water emulsions.
• A waxy & non-waxy rice & waxy maize starch adsorbed at oil droplets, others did not.
• Not related to thermal, crystallinity & molecular properties, amylose, protein.
• Functionality as clean label emulsifier clearly demonstrated.

## Graphical abstract

**Figure:**
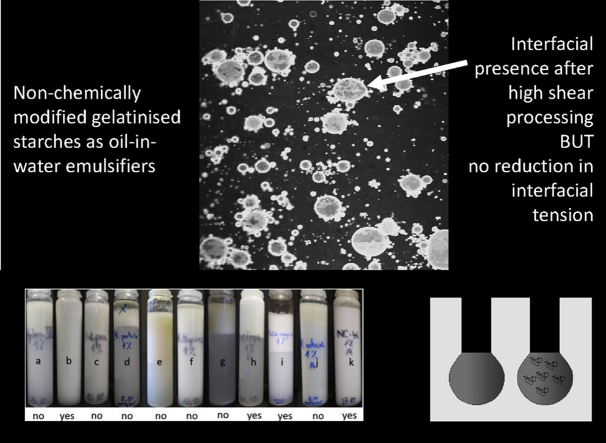

## 1 Introduction

Emulsions are a frequently encountered microstructure in processed consumer goods including foods, paints and cosmetics. They are two-phase liquid dispersions where one phase is present in the form of droplets in another immiscible phase. With the exception of microemulsions they are thermodynamically unstable. Therefore, in practice emulsion microstructures are kinetically trapped through the use of emulsifiers and often also the addition of viscosity enhancers to the continuous emulsion phase. Emulsifiers adsorb at the interface between the two immiscible liquid phases imparting electrostatic or steric barriers between neighbouring and colliding droplets thus preventing coalescence. Adsorption occurs during processing, trapping process induced microstructures if the concentration of added emulsifier is high enough compared to the total interfacial area of the emulsion. Otherwise, coalescence will occur until surface saturation traps the evolving microstructure. Viscosifiers aide the prevention of coalescence by slowing the creaming or sedimentation of individual droplets thus close proximity of droplets which may lead to coalescence.

The research reported here is concerned with emulsifiers suitable to stabilise oil-in-water (o/w) food emulsion interfaces during processing, imparting microstructure stability over storage, but destabilising the emulsion microstructure during oral processing to release in the oil phase entrapped salt or sugar solution. The microstructure of such systems is known as water-in-oil-in-water emulsions generally referred to as multiple, duplex or complex emulsions. The release of the tastant solution from the internal water phase to the vicinity of the taste receptors will impart a higher perceived tastant intensity compared to an emulsion where all of the tastant is present in the external emulsion phase, which we have reported for the case of saltiness enhancement (Chiu, Hewson, Fisk, & Wolf, 2015). The emulsifier was commercial octinyl succinic anhydride (OSA) waxy maize starch destabilising the emulsion during oral processing due to the mechanical actions of tongue and teeth combined with the enzymatic breakdown through salivary amylase. Also applied for emulsion stabilisation have been OSA modified native starch granules (Timgren, Rayner, Sjöö & Dejmek, 2011; Rayner, Timgren, Sjoo & Dejmek, 2012). Preparing our own gelatinised OSA modified waxy maize starch with a degree of modification between 0% and 3%, corresponding to the upper level allowed for OSA modified starch as a food ingredient, we were able to demonstrate that the degree of OSA modification was negatively correlated to tastant perception (Chiu, Tarrega, Parmenter, Hewson, Wolf & Fisk, 2017). However, at less than 2% OSA modification the microstructure of the emulsions was stable only for up to a few days, rendering this approach to reduce salt or sugar in emulsion-based foods commercially almost irrelevant.

Clearly, native starch with no sites of the molecule rendered inaccessible to amylase would provide the fastest digestion kinetics (He, Liu & Zhang, 2008). It is therefore hypothesised to provide best results in terms of taste enhancement. However, to the best of our knowledge there are no literature reports of non-chemically modified gelatinised starch acting as stabilisers of o/w interfaces. There are though literature reports of unmodified native starch granules providing emulsion stability (Li, Li, Sun & Yang, 2013), although to a limited degree. Nevertheless, we revisited this approach. Since, as with any particles applied for interfacial stabilisation of emulsions, smaller starch granules will stabilise smaller droplets, we selected rice starch. More specifically, we selected waxy rice starch to minimise the complexity of the system as the starch component of waxy starches is composed of nearly 100% amylopectin, and not a mixture of amylose and amylopectin. Following our previous experimental protocols, we prepared water-in-oil-in-water emulsions through high shear processing in absence of temperature control. The emulsions were stable against coalescence for several weeks while the granular nature of the waxy rice starch had been lost. This was confirmed by inspecting the droplet surfaces for starch granules with bright field and cross-polarised illumination microscopy. It appeared that the emulsions were stabilised by gelatinised waxy rice starch leading to question whether other commonly applied food starches would show the same functionality. This was investigated in the research reported here utilising starches varying in botanical origin including waxy and non-waxy varieties. A commercial OSA starch was included as control and all of the starches were submitted to a simple oil-in-water (o/w) emulsion assay. Irrespective of whether stable emulsions were formed the starches were extensively characterised to develop an understanding of why (some) non-chemically modified gelatinised starches may stabilise o/w emulsion interfaces. This is of considerable practical interest because native starches are clean label ingredients.

## 2 Materials and methods

### 2.1 Materials

#### 2.1.1 Starches

Table 1 provides an overview of the starches tested in this research for their ability to stabilise o/w emulsion interfaces. Also included are their amylose and protein content as well as their thermal properties in excess water (data acquisition methods described in the following). The starches were used as received.

**Table 1:** Starches.

Starch	Moisture (%)	Amylose (%)	Protein (%)	ΔH (J/g)	T _onset_ (°C)	T _peak_ (°C)	T _endset_ (°C)
OSA starch (NC46 Creamer)	6.0 ± 0.2	4.9 ± 0.4	0.28 ± 0.01	–	–	–	–
High amylose maize (HylonVII)	11.6 ± 0.1	69.2 ± 2.2	0.54 ± 0.02	1.9 ± 0.1	67.8 ± 1.3	77.0 ± 0.3	86.0 ± 3.0
Non-waxy rice	11.1 ± 0.3	31.6 ± 1.3	1.01 ± 0.02	10.7 ± 0.7	70.2 ± 0.1	75.4 ± 0.1	80.7 ± 0.2
Pea starch	12.3 ± 0.2	37.8 ± 1.3	0.26 ± 0.01	10.9 ± 0.5	55.8 ± 0.5	62.2 ± 0.1	70.5 ± 1.1
Potato	13.2 ± 0.4	29.6 ± 1.2	0.14 ± 0.01	13.8 ± 0.3	58.0 ± 0.1	62.1 ± 0.1	68.6 ± 0.2
Regular rice	10.5 ± 0.1	22.0 ± 3.5	0.34 ± 0.03	10.2 ± 0.7	62.6 ± 0.0	69.7 ± 0.0	77.3 ± 0.1
Tapioca	11.8 ± 0.1	25.6 ± 0.6	0.02 ± 0.01	12.3 ± 0.1	63.2 ± 0.3	69.2 ± 0.6	78.0 ± 0.4
Waxy maize AMIOCA TF	10.7 ± 0.3	6.4 ± 0.2	0.24 ± 0.00	12.8 ± 0.2	66.0 ± 0.2	71.9 ± 0.2	78.6 ± 0.2
Waxy maize PRIMA600	11.4 ± 0.1	1.2 ± 0.3	0.06 ± 0.00	9.7 ± 0.0	56.6 ± 0.1	62.0 ± 0.1	70.0 ± 0.0
Waxy rice	13.0 ± 0.4	6.1 ± 0.1	0.66 ± 0.03	12.5 ± 0.8	60.1 ± 0.3	67.5 ± 0.1	74.6 ± 0.3
Wheat	11.4 ± 0.2	32.7 ± 1.1	0.32 ± 0.01	9.3 ± 0.1	55.6 ± 0.0	60.4 ± 0.0	67.3 ± 0.2

#### 2.1.2 Chemicals

Iodine for the amylose assay and sodium azide as antimicrobial agent were purchased from chemicals suppliers (Fisher Scientific, Loughborough, UK, and Sigma Aldrich, Gillingham, UK, respectively). Sunflower oil, serving as the dispersed emulsion phase, was purchased in a local supermarket (supermarket own brand, Sainsbury's, Nottingham, UK). Dimethyl sulfoxide (DMSO) as solvent for starch, Rhodamine B to fluorescently stain starch and Nile Red to fluorescently stain oil were also purchased from a chemicals supplier (Sigma-Aldrich, Steinheim, D). Fast Green to fluorescently stain protein was purchase from Merck (Darmstadt, D)All chemicals were used as received. Water used in this research was milli-Q water.

### 2.2 Starch characterisation

#### 2.2.1 Moisture content

The moisture content of the starches was determined using a moisture analyser (MB90, OHAUS Europe GmbH, Switzerland). 0.57–0.62 g of starch were weighed into an aluminium pan that was then placed into the equipment fitted with a thermogravimetric balance. The sample was dried at 120 °C and the results are presented in Table 1 in percent wet weight basis.

#### 2.2.2 Amylose content

The starches were analysed for their amylose content using a colorimetric assay following a modified published protocol (Martinez & Prodolliet, 1996). 5 mg of starch were mixed with 1 mL of 90% DMSO followed by incubation at 95.0 °C for 1 h. Afterwards, the sample was diluted with 3 g/L iodine in 90% DMSO at a ratio of 1:1, and then 10 times diluted with water. Finally, absorbance at 635 nm was measured using a UV–Vis spectrophotometer (Genesys 10S, Thermo Scientific, Loughborough, UK). Amylose content has been reported in Table 1 as percentage as is.

#### 2.2.3 Protein content

Protein content was estimated by measuring nitrogen by standard combustion method (AOAC, 2000). 5–6 mg of starch was weighed in aluminium crucibles and burned in furnaces at 900 °C/1060 °C, using a CHNS—O Analyser (Flash 2000, CE Instruments Ltd, UK) (AOAC, 2000). The instrument performed with a limit of detection at 0.49 μg and a limit of quantification at 0.70 μg for nitrogen. Sulphanilamide (cert. no.: 183407, CE Instruments Ltd, UK) was used as an external standard. The content of crude protein was calculated by multiplying N by 6.25. The results are reported in Table 1 as percentage as is.

#### 2.2.4 Thermal properties

The thermal properties of the starches in excess water were analysed with differential scanning calorimetry (DSC823, Mettler Toledo, Leicester, UK). Each starch was mixed at a ratio of 1:3 with water for 20 h. Approximately 5 mg of starch dispersion were then weighed into stainless steel pans (40 μl volume, Mettler Toledo, Leicester, UK). The pans were hermetically sealed, placed into the calorimeter and temperature ramped up from 5 °C to 90 °C at a rate 10 °C/min. An empty pan was used as reference. The data traces were analysed for onset temperature (T_onset_), peak temperature (T_peak_), endset temperature (T_endset_) and enthalpy of gelatinisation (ΔH (J/g) utilising the equipment's software (STARe). Samples were analysed in duplicate and results (mean and standard deviation) have been included in Table 1 as g water/g starch.

In addition to the starches successfully stabilised emulsions were submitted to the same analysis, omitting the dilution step with water, to quantify the changes in the thermal properties of the starches due to the emulsification process.

#### 2.2.5 Starch crystallinity

The crystallinity of the starch was characterised using X-ray diffraction. The X-ray diffractometer (Bruker AXS D5005, Bruker AXS, Coventry, UK) equipped with a copper tube operating at 40 kV and 40 mA produced Cu Kα radiation of 0.154 nm wave length with a rotation speed of 60 rpm. The samples were examined at 20 °C over the angular range of 3θ to 38θ.

#### 2.2.6 Molecular weight properties

Molecular weight properties of the starches were assessed by means of their sedimentation coefficient distribution (Harding, Adams & Gillis, 2016), popularly used to describe other large macromolecular assemblies as diverse as seed globulins and ribosomes. The matrix free (i.e. not requiring separation columns or membranes) method of sedimentation velocity in the analytical ultracentrifuge was used to generate these distributions, and comparisons were made at the same loading concentration (0.25 mg/mL) to minimise concentration dependent effects due to hydrodynamic non-ideality (Harding et al., 2016).

Solubilised OSA starch, gelatinised starch and starch contained in the serum phase of stable emulsions was analysed for sedimentation velocity using an Optima XLI Analytical Ultracentrifuge (Beckman Instruments, Indianapolis, USA). Measurement conditions were 25 000 rpm and 20.0 °C. Data were analysed using the least squares ls-g*(s) method with SEDFIT (Dam & Schuck, 2004; Harding, 2005; Harding, Adams, Almutairi, Alzahrani, Erten, Kok & Gillis, 2015). All distributions were normalised to a maximum value for g(s) = 1 for the main peak, at a range of sedimentation coefficient from 0 to 500 Svedberg (S), where 1 S = 10 ^−13^ s. Based on previous amylopectin in water hydrodynamic data (Lelievre, Lewis, & Marsden, 1986; Ahmad, Williams, Doublier, Durand, & Buleon, 1999; Harding et al., 2016) the approximate molecular weight in Da (≡ g/mol) of resolved components in the distribution was estimated from their sedimentation coefficient according to the following formula:

(1) $$M=3100\;s^{1.75}$$

This formula derives from the power law or scaling relation

(2) $$s=K_{s}M^{b}$$

(Tsvetkov, Eskins, & Frenkel, 1970; Harding, Varum, Stokke & Smidsrød, 1991; Harding et al., 2015) and previous amylopectin in water hydrodynamic data (Lelievre et al., 1986; Ahmad et al., 1999; Harding et al., 2016). The power law coefficient b is obtained from the Tsvetkov (Tsvetkov et al., 1970) relation

(3) $$b=\frac{2-a}{3}$$

where *a* is the power law coefficient from the corresponding equation to (1) linking intrinsic viscosity with molecular weight. *a* has been found to be 0.31 (Colonna, Doublier, Melcion, Demonredon & Mercier, 1984) and ∼0.29 (Millard, Dintzis, Willett & Klavons, 1997) and so this can reasonably taken to be ∼0.30, leading to a value of *b* = 0.57. o find κ_s_ Equation (1) can be used for known values of *s* and its corresponding *M*. Using the value of 280 S and its corresponding value of 6 × 10^7^ (Banks, Geddes, Greenwood & Jones, 1972) leads to κ_s_ = 0.0103 and Equation (1). Molecular weights that have been assigned to any peaks that are predominantly due to amylose will of course be in error to some degree.

The serum phase of stable emulsions was recovered using a syringe (500 μL gas tight syringe, with an outer diameter of needle 0.5 mm, SGE Analytical Science, US), followed by dilution with water prior to analysis.

### 2.3 Emulsion methods

#### 2.3.1 Emulsion preparation

Starches were screened for emulsifying ability by processing o/w emulsions containing 20% oil and 1% starch (weight by weight on total emulsion). The remainder was water containing 0.02% sodium azide to prevent microbial spoilage of the emulsions. In each case 100 g of emulsion were prepared through processing with a high shear overhead mixer (LM5 fitted with emulsor screen, Silverson, Chesham, UK) applying a two-step process; starch gelatinisation followed by emulsifying the oil. Initially, the appropriate amount of starch and water were weighed into a tall form 200 mL glass beaker and processed at 8000 rpm for 5 min. During this process the temperature increased from ambient (around 20 °C) to between 60 and 70 °C. Afterwards, without interruption, 20 g of oil were added followed by further mixing at 8000 rpm for 5 min during which temperature increased further. Final temperatures varied between 75 and 85 °C. The temperature profile the starches experienced during sample preparation is illustrated exemplary in Table 2 for all of the trials based on waxy rice starch reported here. The upper end of which is above T_endset_ for all starches with the exception of the high amylose maize starch (HylonVII), see Table 1.

**Table 2:** Temperature profile during emulsion preparation reported for waxy rice starch as representative example for all of the starches. Data for three repeat experiments are shown for each concentration of starch applied.

Process step	Process time (min)	Temperature (°C)											
		1% starch			2% starch			3% starch			4% starch		
waxy rice starch + water 8000 rpm/5 min	0	23	22	21.4	22.9	22.4	23.1	23	22.7	22	22.4	21.3	25
	1	31	29	29.8	31	30	30.5	31	30.7	30.6	34.4	29.6	33.4
	2	40.2	37	39	40.2	39.8	39.1	41	40	39.9	43.9	39.9	42.8
	3	48.9	47.6	47.9	48.7	48.7	47.7	48.6	48.9	48.7	53.9	48.4	50.3
	4	56.9	54.7	55.7	57	57	55.2	55.7	56.7	56.5	61.9	55.7	57.3
	5	64.7	62.7	63.2	64.4	64	59.9	62	64.6	64	68.7	62.8	64.3
addition of oil 8000 rpm/5 min	0	61	60	60	61.1	60.6	59	59.9	61.4	60.6	60	58	59.6
	1	66	64	65	65	64.9	63	63.3	64.8	64.3	65.2	61.5	62.7
	2	70.9	69.5	70.3	69.1	68	66	66.5	68.2	68	69.9	64.7	68
	3	74	72.8	73.9	72.7	71.6	69.3	70.3	72.6	72	74.7	69	71.9
	4	77	75.3	78	76	73.1	72.3	74.7	77.6	76.4	80	73	75.7
	5	80	78	80	80	75	76	76.7	80.7	80	85.4	77	80

Following processing the emulsions were transferred into 50 mL tall capped glass vials and stored at 20 °C for visual assessment of creaming and coalescence. A yellowish appearance of the cream layer, clearly visible large oil droplets, i.e. with a diameter of plus 1 mm, and/or the formation of a bulk oil layer on top of the cream phase were taken as signs of coalescence. Starches applied in emulsions showing coalescence were identified as non-emulsifying. Non-emulsifying starches were then re-evaluated at 2, 3 and 4% of starch to validate their classification as non-emulsifying. Emulsifying starches were also applied at these higher concentrations, with one exception mentioned in the results section, to assess impact on droplet size and other emulsion properties as described in the following.

#### 2.3.2 Microstructure

To validate droplet size distribution data and to assess droplet flocculation, emulsion microstructure was visualised using bright field illumination on a digital microscope (EVOS fl, Life Technologies, Paisley, UK). Samples were prepared by placing a drop of emulsion onto a glass slide followed by carefully sliding over a glass cover slip.

Confocal laser scanning microscopy was applied to validate the presence of starch and lack of the presence of protein at the o/w interface. A drop of emulsion was placed onto a glass cover slide, carefully mixed at a ratio of 1:2 with Rhodamine B solution (Sigma Aldrich, 0.01 g Rhodamine B/1 L water), Fast Green solution (Merck, 0.1 g/L in water) and Nile Red (Sigma Aldrich, 0.01 g/L in polyethylene glycol) using a pipette tip followed by sliding over a glass cover slip. Nile Red was used to stain lipid, Fast Green to stain protein and Rhodamine B to stain starch. The sample was then imaged with a confocal laser scanning microscope equipped with an Argon laser (488 nm excitation wavelength) and HeNe laser (561 and 633 nm excitation wavelength; Zeiss LSM880, Carl Zeiss Microscopy GmbH, Jena, Germany). A band pass filter between 580 and 620 nm was selected for the detection of Nile Red; when excited at 488 nm, and 651–710 nm for the detection of Fast Green; when excited at 633 nm. A band pass filter for Rhodamine B was selected between 550 and 580 nm and excited at 561 nm. Images were acquired using an ×60 objective.

#### 2.3.3 Droplet size

Droplet size distributions of the o/w emulsions were acquired using a low angle diffraction particle analyser (LS 13 320, Beckman Coulter, High Wycombe, UK) fitted with a liquid dispersion cell (Universal Liquid Module) containing water. Appropriate amount of emulsion, as indicated by the equipment software, was added to the water before measurement commenced. Three diffraction patterns were averaged and analysed by the equipment software based on the refractive index of 1.333 for water, as the continuous dispersion phase, and 1.54 for the material adsorbed at the surface of the oil droplets following a published method (Sjoo, Emek, Hall, Rayner & Wahlgren, 2015). Results are presented as surface area based droplet size distributions. Ignoring the very small third population at much larger droplet diameters for a couple of the emulsions, the distributions were bi-modal and thus two Sauter diameters are reported for distribution. These were obtained by splitting the area under the curve into two either at the minimum between the two area based distribution peaks or where the shoulder ended or started.

#### 2.3.4 Interfacial tension

A drop shape tensiometer (PAT-1, Sinterface, Berlin,D) was used to assess interfacial tension at the oil/water interface where the water phase was pure water, water containing 4% OSA starch or 4% gelatinised waxy rice starch. The starch dispersion was obtained by mixing starch with water at 8000 rpm for 10min using the same high shear overhead mixer as for emulsion processing. The sample was then allowed to cool to room temperature (approx. 20 °C) by placing onto the lab bench before being applied to the syringe of the drop shape tensiometer. This syringe was fitted with a straight stainless steel capillary with an outer diameter of 2 mm and used to dose a pendant water drop of 25 mm^3^ volume into the oil phase contained in a quartz glass cuvette. Droplet volume was held constant during the measurement and all measurements were conducted at 20 °C.

Acquisition of interfacial tension data at 20 °C was representative of our observations with regard to the stabilisation of emulsions at much higher temperature (75–85 °C). Those starches stabilising emulsions during the high temperature process successfully also stabilised emulsions processed after cooling the gelatinised starch to 20 °C followed by emulsifying in the oil with the same high shear process but maintaining this lower temperature through cooling with ice.

## 3 Results and discussion

### 3.1 Starch stabilised emulsions

#### 3.1.1 Screening of starches

**Fig. 1:**
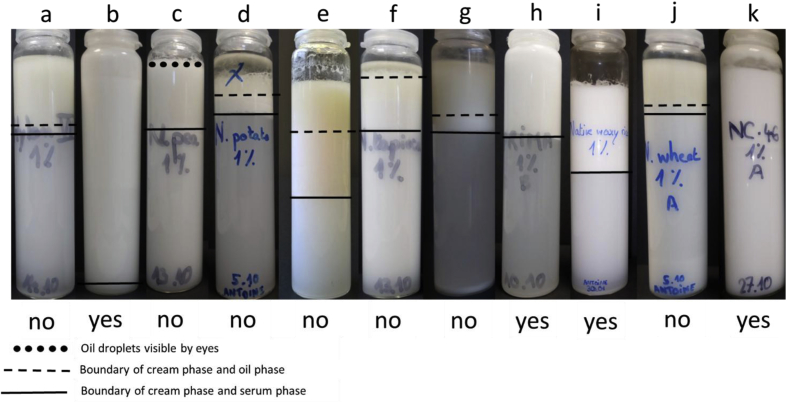
Emulsions processed with (a) high amylose maize (Hylon VII), (b) non-waxy rice, (c) pea, (d) potato, (e) regular rice, (f) tapioca, (g) waxy maize AMIOCA TF, (h) waxy maize PRIMA600, (i) waxy rice, (j) wheat, and (k) OSA-starch NC46 after 1 day of storage. Ability of the starches to stabilise o/w emulsions was judged by eye (large droplets, bulk oil layer) and assessment outcome is indicated by “no” and “yes = can emulsify”. All emulsions contained 1% of starch and 20% of oil and were stored at 20 °C.

All of the starches shown in Table 1 were submitted to the same emulsion processing assay. They were applied at a concentration of 1% to emulsify 20% of oil as it was already known that at this concentration, utilising the same process, the reference OSA starch would stabilise o/w emulsions (Chiu et al., 2015). Ability of the starches to stabilise the o/w emulsions was judged by assessing the emulsions in their storage vials one day after processing, see Fig. 1. If signs of coalescence were noted, see 2.3.1, the respective starch (in their gelatinised form) was classed as non-emulsifying. These included the high amylose maize starch (Hylon VII), the pea starch, the potato starch, the regular rice starch, the tapioca starch, the waxy maize AMIOCA TF and the wheat starch. The non-waxy rice starch, the waxy rice starch, the waxy maize starch PRIMA600 as well as the OSA-starch on the other hand had successfully emulsified the oil phase. Some of the emulsions showed creaming, however, this was not included in the stability criterion. Creaming can be prevented by addition of viscosifiers to the continuous emulsion phase and does not necessarily mean that coalescence has occurred. The non-waxy rice starch stabilised emulsion had the appearance of a viscoelastic emulsion. Additional testing of the non-emulsifying starches at 2, 3 and 4% of starch revealed that only the tapioca starch applied at 4% was also judged to be emulsifying; although in the following it will remain classified as non-emulsifying. Stable emulsions were further analysed.

#### 3.1.2 Emulsion microstructure and droplet size distribution

**Fig. 2:**
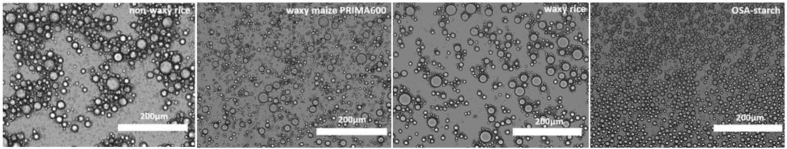
Light micrographs, taken one day after processing, of emulsions containing 20% of oil and 1% of, from left to right, non-waxy rice starch, waxy maize starch PRIMA600, waxy rice starch and OSA starch. Length of scale bar: 200 μm.

Stable emulsions, i.e. those prepared with the non-waxy rice starch, the waxy maize starch PRIMA600, the waxy rice starch and the OSA starch were analysed for microstructure and droplet size distribution. Fig. 2 shows the microstructure of these emulsions prepared at 1% starch as it appeared one day after processing. The images reveal differences in the extent of droplet flocculation, droplet size and polydispersity. The emulsion stabilised with the OSA starch showed no signs of flocculation and the droplets were visibly smaller and less polydisperse compared to the other three emulsions. The emulsion stabilised with the non-waxy rice starch was the most flocculated with a network of droplets that appeared to be sample spanning in congruence with the viscoelastic solid- or gel-like texture of this emulsion. All of the other emulsions behaved much more like a viscoelastic liquid. The two waxy starch stabilised emulsions showed some degree of droplet flocculation, but clearly not a sample-spanning network, hence their liquid-like texture. The waxy rice starch stabilised emulsion appeared to be slightly more flocculated. This may have been the result of the higher amylose content in this starch compared to the waxy maize starch PRIMA600, see Table 1.

**Fig. 3:**
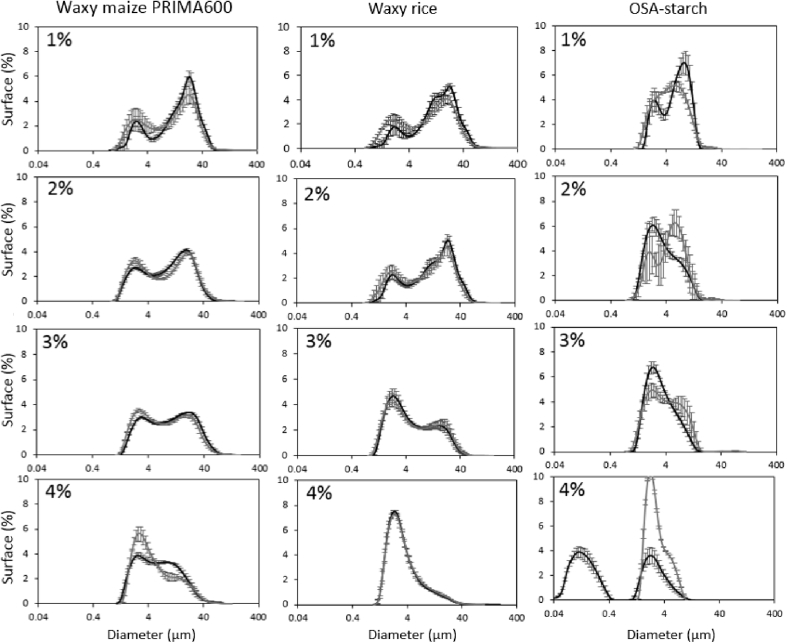
Surface area weighted droplet size distributions of emulsions stabilised with the waxy maize starch PRIMA600, the waxy rice starch and the OSA starch. All emulsions contained 20% of oil and 1, 2, 3 or 4% of starch. Data shown were acquired one day after emulsion processing (black line) and after 3 months of storage at 20 °C (grey line).

With regard to the acquisition of droplet size distributions on the emulsions the non-waxy rice starch stabilised emulsion was excluded from this assay. Sampling the emulsion gel into the liquid dispersion cell proved challenging and results would have been representative of large flocs of droplets or small fragments of emulsion gel instead of primary droplet size. The droplet size distributions of the remaining emulsion systems are presented in Fig. 3 including data at different starch levels acquired one day and 3 months after processing. All of the droplet size distributions were bi-modal although a very small third population can just about be identified for a couple of the distributions. This third population was ignored when determining the Sauter diameters reported in Table 3. Comparison with the micrographs indicates that the two modes of the droplet size distributions could be indeed attributed to two populations of droplets. The range of droplet size in the emulsions was comparable and within what would typically be found in food formulations. The OSA starch stabilised emulsions had smaller droplets compared to the other starch stabilised emulsions. The Sauter diameters remained approximately unchanged over the three months of storage. A slight rise was seen for the Sauter diameters for the emulsions stabilised with 2% or 4% waxy maize PRIMA600 and 4% OSA starch.

**Table 3:** Sauter diameter corresponding to the two populations of each droplet size distribution shown in Fig. 3. The very small third population at higher droplet diameters, which were just about identifiable in Fig. 3, were ignored.

Starch concentration (%)	Sauter diameter (μm)															
	1				2				3				4			
Storage time→ starch type↓	1 d		3 months		1 d		3 months		1 d		3 months		1 d		3 months	
Peak	1	2	1	2	1	2	1	2	1	2	1	2	1	2	1	2
Waxy maize PRIMA600	2.9 ± 0.2	22.8 ± 0.9	3.1 ± 0.5	22.7 ± 1.8	2.9 ± 0.0	18.8 ± 1.0	3.3 ± 0.1	22.4 ± 0.8	3.1 ± 0.0	18.3 ± 0.5	3.6 ± 0.0	20.2 ± 0.6	3.1 ± 0.0	14.5 ± 0.3	4.0 ± 0.1	20.0 ± 1.2
Waxy rice	2.5 ± 0.4	20.8 ± 0.9	2.3 ± 0.3	19.7 ± 1.2	2.7 ± 0.1	21.6 ± 0.1	2.6 ± 0.3	20.8 ± 1.2	3.5 ± 0.1	19.1 ± 1.0	3.5 ± 0.2	19.4 ± 0.9	4.2 ± 1.1	16.6 ± 2.5	3.4 ± 0.1	16.6 ± 1.1
OSA starch	2.8 ± 0.0	8.5 ± 0.1	2.6 ± 0.0	7.2 ± 1.0	2.6 ± 0.1	7.8 ± 1.2	2.2 ± 0.2	6.9 ± 1.0	2.6 ± 0.3	6.9 ± 0.3	2.5 ± 0.1	8.3 ± 0.0	0.2 ± 0.0	3.2 ± 0.0	2.6 ± 0.0	5.9 ± 0.0

#### 3.1.3 Interfacial properties

**Fig. 4:**
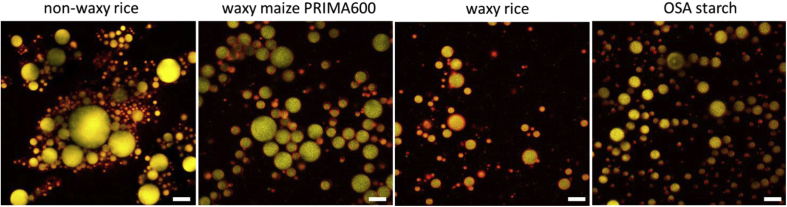
Confocal laser scanning micrograph of an o/w emulsion stabilised with 4% non-waxy rice starch, waxy maize PRIMA600, waxy rice and OSA starch. The emulsions were stained with Rhodamine B, Nile Red and Fast Green to visualise the starch (red), lipid (yellow) and protein (green, not visible), respectively. Length of scale bar: 10 μm.

To develop an understanding of the origin of the emulsifying ability of waxy rice starch the interfacial tension at the o/w interface was measured utilising the pendant drop technique. As described in the methods section preparation of the waxy rice starch for this measurement included high temperature processing as in the case of emulsion processing followed by cooling to room temperature (approx. 20 °C). It was validated that this starch dispersion would stabilise o/w emulsions through high shear processing at 20 °C before interfacial tension measurement at 20 °C. In the absence of any starch interfacial tension asymptotically reached a value of 26.5 ± 0.4 mN/m after a slightly decreasing trend for the first 120 s due to the presence of naturally present surface-active molecules in sunflower oil. The data traces overlapped with the data traces acquired in presence of gelatinised waxy rice starch in the aqueous phase; in this case the averaged value after levelling off was 26.8 ± 1.3 mN/m. However, in presence of OSA starch in the aqueous phase, following an initial longer decrease, the interfacial tension levelled off at 18.2 ± 0.1 mN/m. OSA starch has been created to be interfacially active and a decrease in interfacial tension was expected. However, it appears that gelatinised waxy rice starch did not adsorb at the interface during the interfacial tension analysis leading to the conclusion that it is not interfacially active. As this starch was still stabilising o/w emulsions it was hypothesised that, during high shear processing, molecules or molecular aggregates of starch would contact the o/w interface and then adhere to it imparting the observed emulsion stability. The presence of starch at the o/w interface was validated by confocal laser scanning microscopy; see Fig. 4. The red domains in Fig. 4 indicate starch-rich regions and these are clearly located at the droplet interfaces. Protein was not visible in these micrographs which is not surprising given the low amount of protein in any of these starches (see Table 1).

### 3.2 Starch characteristics and relationship to emulsifying ability

#### 3.2.1 Thermal properties

Thermograms of all of the starches acquired in excess water showed a single endothermic peak. The onset, peak and endset temperature of the transition from granular to gelatinised starch and enthalpy of this phase transition were reported in Table 1. The temperature data show that the final emulsion processing temperature of between 75 and 85 °C was higher than the peak temperature of the phase transition from granular to gelatinised starch for all of the starches. Hence, all of the starches were to a large extent gelatinised at the end of the emulsification process. It appears that differences in emulsification ability cannot be explained by lack of gelatinisation. The gelatinisation enthalpy values bear no significance to the emulsifying ability but were included in Table 1 as part of the thermal property characteristics of the starches applied in this study.

The thermograms acquired on the processed emulsions stabilised with the non-waxy rice starch, the waxy rice starch and the waxy maize starch PRIMA600, lacked an endothermic peak indicative of the fully gelatinised state of the starch in the system.

#### 3.2.2 Crystallinity pattern

**Fig. 5:**
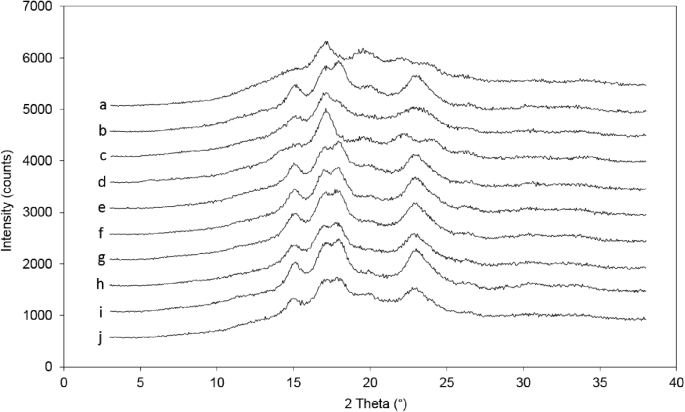
XRD pattern of (a) high amylose maize starch (Hylon VII), (b) non-waxy rice starch, (c) pea starch, (d) potato starch, (e) regular rice starch, (f) tapioca starch, (g) waxy maize starch AMIOCA TF, (h) waxy maize starch PRIMA600, (i) waxy rice starch and(j) wheat starch. Data have been offset for clarity of presentation.

The crystallinity pattern of each starch was analysed with X-ray diffraction to inspect whether amylose-lipid complexes ought to be considered in explaining the emulsifying ability of some but not other starches included in this study. Amylose-lipid complexes can be naturally present in native starches (Evans, 1986) and form during gelatinisation in the presence of lipids. In their unordered form, amylose-inclusion complexes are known as “type I” complexes while the amorphous complexes, organized in lamellae, are referred to as “type II” amylose-inclusion complexes (Putseys, Lamberts & Delcour, 2010). In XRD patterns these inclusion complexes are identified as V-type polymorph, with diffraction peaks at 2 θ of around 12.5° and 20.0° (Takeo, Tokumura & Kuge, 1973) for native starches. The XRD patterns acquired on all of the starches applied in this study are presented in Fig. 5; the intensity of the V-pattern varied depending on the starch origin. The greatest intensity was shown by the high amylose maize starch (Hylon VII). The V-pattern intensity was greater for the non-waxy rice starch compared to the waxy rice starch due to the comparatively higher amount of amylose in the non-waxy variety. Similarly, in the case of the waxy maize starch PRIMA600 and AMIOCA TF, the intensity of the V-pattern was diminished. So, as expected, the XRD patterns reflect the level of amylose in the starches which was already suggested to not be indicative of their emulsifying ability. However, the question whether amylose-lipid complexes do form in these systems during processing and therefore contribute to emulsion stability requires further analysis, e.g. based on nuclear magnetic resonance (Beeren, Meier & Hindsgaul, 2013).

#### 3.2.3 Sedimentation coefficient distribution

**Fig. 6:**
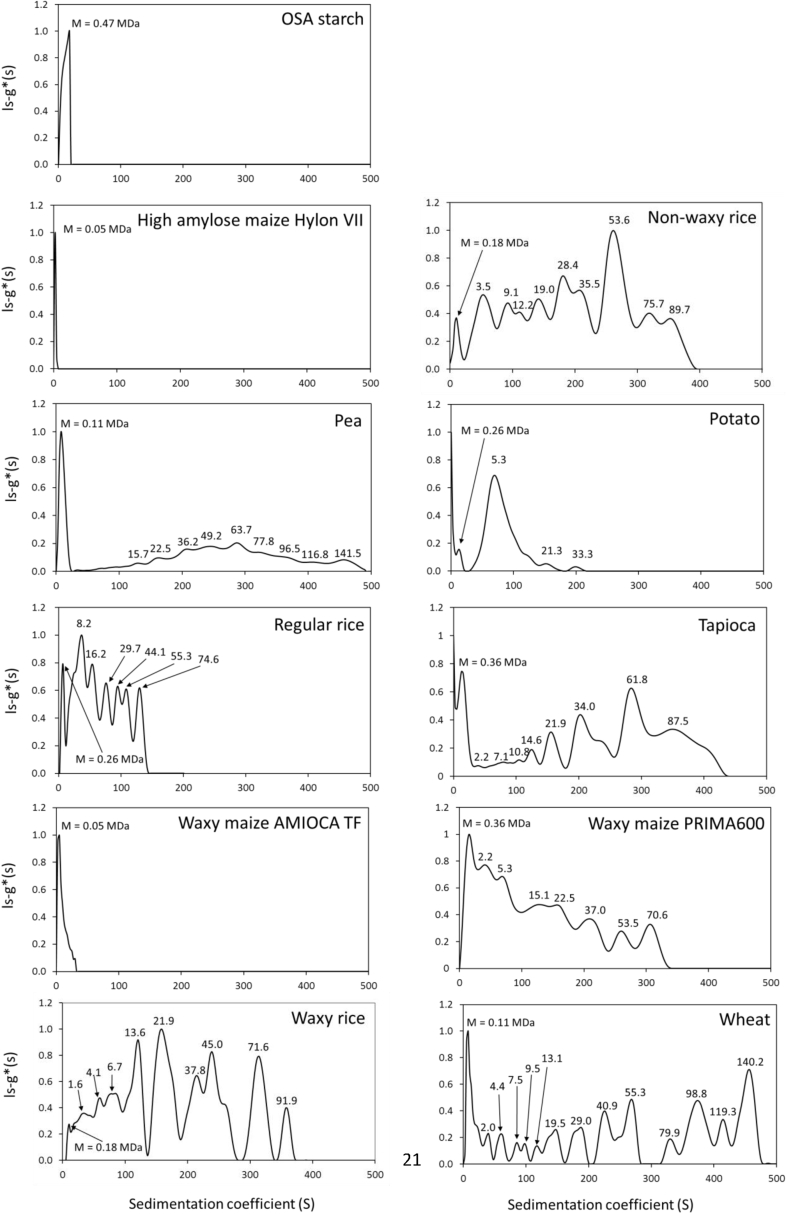
Sedimentation coefficient distributions (at a loading concentration of 0.25 mg/mL) and corresponding molecular weight (in MDa) estimated for each component peak of all starches as they would be in aqueous dispersion before addition of oil for emulsification.

**Fig. 7:**
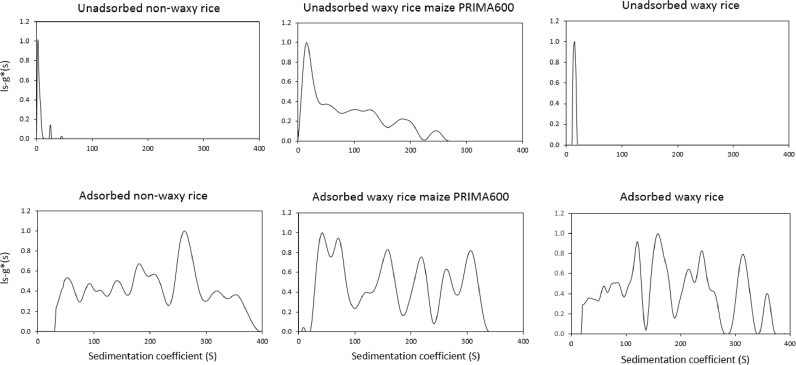
Sedimentation coefficient distributions of unadsorbed starch fraction (top row) and interfacially adsorbed starches (bottom row). The bottom row data were obtained by subtracting the top row data from the gelatinised starch data presented in Fig. 6. Other conditions as in Fig. 6.

The sedimentation coefficient distributions for all starches, as they were present in aqueous dispersion prior to the addition of oil in the emulsification process are presented in Fig. 6. The results shown in Fig. 7 indicate which fraction of the emulsifying starches adsorbed at the o/w interface. These data were generated by carrying out additional ultracentrifugation analysis on the aqueous phase of these emulsions. The sedimentation coefficient distribution of the starch fraction that adhered to the interface during processing was then calculated by subtracting this data from the data obtained for the starch prior to emulsification. This is a first approximation as starch may have associated with the adsorbed starch which creams with the emulsion droplets and is thus not captured as fraction of the serum phase.

It is worth stressing here that the sedimentation coefficient distribution data are representative of the starch processed in aqueous environment exactly the way as the emulsions in terms of shear, time and temperature. Water is not a good solvent for starch and therefore the data do not relate to individual starch polymers but to aggregates of starch polymers as confirmed by the multimodal nature of the distributions.

The results presented in Fig. 6 reveal a range of sedimentation coefficient distribution patterns. The OSA starch, analysed as the commercial emulsifying reference starch, showed a single peak with a maximum at ∼17.7 S. The three non-chemically modified starches identified as emulsifying starches (non-waxy rice starch, waxy maize starch PRIMA600, waxy rice starch) showed a broad sedimentation coefficient distribution characterised by several peaks up to an *s* of ∼400 S. The high amylose maize starch Hylon VII, the potato starch and the regular rice starch, all three not showing emulsifying functionality, had narrower sedimentation coefficient distributions at the lower end of the Svedberg unit range (<∼200 S), typically with fewer peaks. The pea starch, also not emulsifying, had a high peak in the lower Svedberg unit domain with a broad low intensity population covering the Svedberg unit range up to 500 S with a small number of peaks that were not isolated from each other. The wheat starch, again not emulsifying, showed a similar pattern although with a larger number of isolated peaks or populations in the mid to high Svedberg unit domain. The sedimentation coefficient distribution of the tapioca starch, which stabilised the o/w emulsion processed in this study when applied at 4%, appeared similar to that of pea starch but with larger populations at the higher Svedberg unit domain.

Since it was already known which starches showed emulsifying ability the sedimentation coefficient distribution data were assessed if a certain fraction of starch polymers or polymer aggregates at the higher size (sedimentation coefficient, in Svedbergs) range was required for starches to show this functionality. The distributions are shown in Fig. 7 and in Table 4 the proportion of material with sedimentation coefficients >50 S is compared to with emulsifying ability. From these it seems that emulsifying ability appears to show no clear dependence on size, which is an important find.

**Table 4:** Emulsifying ability of starches and proportion of material with sedimentation coefficient >50 S.

Starch	Proportion (%) of material > 50 S	Emulsifier
OSA starch (NC46 Creamer)	0	Yes
High amylose maize (HylonVII)	0	No
Waxy maize AMIOCA TF	0	No
Waxy maize PRIMA600	73	Yes
Pea starch	78	No
Wheat	85	No
Tapioca	86	No
Potato	86	No
Regular rice	87	No
Waxy rice	91	Yes
Non-waxy rice	92	Yes

## 4 Conclusions

It can be concluded that at least three non-chemically modified starches exist that will stabilise oil-in-water emulsions processed under high shear as long as the starches gelatinise during processing. This ability to act as emulsifier through interfacial adsorption during processing was not related to amylose content or crystallinity pattern. Characterising the molecular characteristics of the starches and the fraction that remained in the emulsion serum phase with the method of analytical ultracentrifugation (AUC) also failed to increase understanding of criteria for emulsifying ability. Nevertheless, this research has, to the best of our knowledge, for the first time shown that waxy rice starch, non-waxy rice starch and waxy maize starch PRIMA600 act as emulsifiers. So, there are natural or clean label starches, not only alternative to OSA starch but also to other synthetic food emulsifiers, that can be applied for the interfacial stabilisation of emulsion based foods.

## Appendix A Supplementary data

mmc1 mmc1
